# Does Dog Ownership Affect Physical Activity, Sleep, and Self-Reported Health in Older Adults?

**DOI:** 10.3390/ijerph16183355

**Published:** 2019-09-11

**Authors:** Eliška Mičková, Kristýna Machová, Klára Daďová, Ivona Svobodová

**Affiliations:** 1 Department of Ethology and Companion Animal Science, Faculty of Agrobiology, Food and Natural Resources Czech University of Life Sciences, Prague 165 00, Czech Republic; mickova.eliska@seznam.cz (E.M.); Svobodovai@af.czu.cz (I.S.); 2 Faculty of Physical Education and Sport, Charles University, Prague 162 52, Czech Republic; dadova@ftvs.cuni.cz

**Keywords:** older adults, dog ownership, physical activity, health

## Abstract

Physical activity (PA) is crucial for maintaining good health of older adults and owning a dog and walking it can enforce it. The purpose of this study was to evaluate the effect of dog ownership on PA in older adults as well as its positive impact on perceived degree of health, and sleep. There were 44 participants of mean age 68 ± 5.4 years (18 males, 26 females) enrolled in this study (dog owners—DO, n = 26; non-dog owners—NDO, n = 18). Xiaomi Mi Band 2 accelerometer, International Physical Activity Questionnaire- Short form (IPAQ-Short Form) and SF-36 questionnaires were used to measure the level of PA, sleep, and subjective health. A statistically significant difference was observed in favor of dog owners in most of the monitored parameters. All accelerometer PA parameters (step count, activity time, distance, calories) showed a significant difference at a *p* < 0.01. Sleep parameters were significant in total sleep length (*p* = 0.05) and light sleep length (*p* < 0.05). DO reported higher total PA time (min/week), MET/min/week spent in walking, and spent calories/week (*p* < 0.05). In SF-36 they reported higher score (*p* < 0.05) in general health, physical functioning, social functioning, pain, vitality, and emotional well-being. Body mass index (BMI) was significantly lower in the DO group (*p* < 0.01). The results suggest that dog ownership may affect the overall PA and health of older adults.

## 1. Introduction

One of the factors increasing mortality rates in the world is physical inactivity of which around 3.2 million (6%) people die every year [1]. As the average age of the population increases, there is simultaneously growing number of older adults who, due to naturally deteriorating health with age, engage in physical activity less than the young generations [2]. Decreased physical activity (PA) affects incidence of pathologic conditions like stroke, heart attack, diabetes, and asthma [3]. Another aspect related to lower level of physical activity is increased sedentary time. People aged 70–79 spend up to 9.6 h sitting every day [4], which may result in muscle weakness, loss of muscle mass, and storing of excess body fat [5].

At least 150 min of moderate physical activity (MPA) per week or at least 75 min of vigorous physical activity (VPA) per week is recommended to maintain good health for people over 65 years of age. An appropriate combination of moderate and intense physical activity is also an option. Such activities should be spread throughout the week [6].

According to Mace et al. [7] up to 65% of older adults over 65 years do not achieve the recommended levels of physical activity. Some older adults do not perform any PA, although even a low level of moderate and vigorous physical activity has a positive effect on health and reduces mortality rate by 20–22% [8,9].

Owning a dog and walking it seems to be a pleasant activity for some people, which can lead to increased physical activity. According to Feng et al. [10] older adults owning a dog are up to 12% more active than their peers who do not own one. If the dog shares a household with an older adult, then there is a 23% lower probability of sitting for more than 8 h per day [11]. Owning a dog is a possible solution for lonely older adults, for whom owning a dog means having a companion; the result is that they suffer less from conditions like depression and that they have to take responsibility for the dog [12]. Dog ownership has a positive effect on physical activity as well as on mental health of older adults. Mental health is closely related to physical activity and PA is an important part of comprehensive rehabilitation. Dog ownership offers a way to make older adults more active and give them necessary social interaction [13].

Deteriorating quality of sleep is also a problem associated with older age. Many older adults suffer from symptoms of insomnia, of which around 37% have difficulties falling asleep at night and nearly 29% of them report intermittent sleep during the night [14]. Sleep quality is also one of the areas that physical activity largely affects. One way of improving the quality of sleep could be to own a dog and being required to walk it. A positive effect of PA on subjectively measured sleep (shorter sleep onset latency, longer rapid eye movement sleep latency) was found in all age categories [15,16]. Specifically, older adults with high PA experience an increase in overall sleep time, sleep efficiency, improved sleep quality, and a lower incidence of sleep disorders [17]. Some dog owners have a very close relationship with their pets and allow them to sleep with them in bed. Evidence of the canine’s influence on sleep quality when present in bed, is inconsistent [18]. However, it was observed that the owners feel more secure and satisfied when falling asleep. Impact of dog ownership on the sleep of older adults is not yet fully explored. This research focuses in detail on the level of physical activity of older dog owners and non-dog owners as well as on sleep differences between the two groups and their subjectively measured quality of life.

The aim of this study was to see how dog ownership affects physical activity, sleep, and self-reported health in our group of older adults.

## 2. Materials and Methods

### 2.1. Participants

There were 44 older adults enrolled in this study. In the first group (dog owners, DO) there were 26 participants (14 males; 12 females), in the second group (non-dog owners, NDO) there were 18 participants (four males; 14 females). The mean age was 69.5 ± 5 years.

The inclusion criteria consisted of being 60–79 years old, ability to fill in the required questionnaire, willingness to wear an accelerometer throughout the specified period, ability to perform activities of daily living (ADL), and absence of serious impairment of cognitive functions beyond the standard development. Dog ownership was also an inclusion criterion for participants in the first group.

### 2.2. General Procedures

Data were collected for 16 months—from December 2017 to March 2019—in the territory of the Czech Republic. Accelerometers were distributed by students who received proper training in the use of the device and were familiar with the methodology of research as well as the collection of data and the use of the respective technology. They were also consistently instructed on how to provide tutorials for older adults and how to correctly fill in the International Physical Activity Questionnaire (IPAQ- Short Form) and the 36-Item Short Form Survey (SF-36) questionnaires to evaluate the respondent’s state of health.

Only those owners within the DO group who had said that they walked their dog regularly (at least twice a day) were selected. Participants were excluded if their dogs dwelled in the garden and the owner did not walk it. The size of the dog breed was not an excluding and limiting criterion. Within the NDO group, there were older adults who did not own or share a house with a dog.

All participants signed informed consent where they agreed to be enrolled in the study, to use of the information provided in the questionnaires, and the information sourced from the accelerometers. The study was approved by the Institutional Review Board of the Czech University of Life Sciences, Prague and Ethics Committee of Czech University of Life Sciences Prague.

### 2.3. Measurement

#### 2.3.1. Accelerometer

All participants were given an accelerometer (Xiaomi Mi Band 2, Anhui Huaomi Information Technology Co., Ltd.; Hefei, China) which measured the number of steps. It also recorded the time the participants spent performing physical activity as well as the distance they covered, and the number of calories burned, the total sleep time, the deep sleep time, the light sleep time, and the time when the participant is awake at night. To store the measured data, the device needs to be synchronized with Mi Fit—an application that is compatible with accelerometers that we used. All data that the accelerometer recorded for individual research participants were uploaded to the application, which allowed them to be merged after the end of each monitored period. Xiaomi Mi Band 2 was chosen because it is cheap, accessible, and easy to operate [19]. It can reliably measure number of steps, distance, and sleep duration, but measuring energy consumption (calories) is inadequate and it must be noted when evaluating acquired data [20].

Participants wore the accelerometer continuously for nine consecutive days. Data were divided into 24-h periods. Data from the first and the last day were discarded as they did not represent the full 24-h period. Sleep periods were used only in relation to the measured daily activity. Thus, seven 24-h periods from seven days and seven consecutive nights were used for the evaluation.

#### 2.3.2. IPAQ

The IPAQ (International Physical Activity Questionnaire) was administered to summarize the levels of intense and moderate PA, walking time, and sedentary time in the last seven days [21]. The evaluation of the physical activity level was done by applying the short form in Czech version (CKV UP, 2006), in which respondents were asked to report the number of days and the duration of the vigorous (V), moderate (M), walking activity (W), and a combined total physical activity score. This short version has demonstrated an acceptable test-retest reliability and criterion-related validity in a 12-country evaluation study [21].

All scores were expressed in MET-minutes/week (www.ipaq.ki.se). For the analysis of IPAQ data IPAQ guidelines were followed [22].

The short form was chosen for its less time-consuming feature as well as for containing all monitored data. The questionnaire also includes demographic data such as gender, age, and number of years of education. In order to calculate the BMI, the questionnaire was supplemented by asking about their height and weight. According to WHO instructions, respondents were categorized into the following categories: normal, 18.5–24.9; grade 1 overweight, 25–29.9; grade 2 overweight, 30–39.9; and grade 3 overweight, >40 [23]. As presented in different studies, completing the questionnaire could be distorted because of not being able to remember the activity that was performed, so it is advisable to use accelerometers for data collection and to use the data from the questionnaire as additional values [24].

#### 2.3.3. SF 36

The SF 36 questionnaire was used to describe subjectively perceived degree of health of respondents in the last 4 weeks. The questionnaire contained a multi-level scale that evaluates eight health concepts. These were physical functioning, the role of limitations due to physical health, social functioning, pain, emotional well-being, the role of limitations due to emotional health, vitality, and general health. The questionnaire also evaluated any change in health in the past year [25]. According to Ware et al. [25] reliability coefficients have consistently exceeded recommended standards for group level analysis.

### 2.4. Data Analysis

All data were analyzed using STATISTICA (StatSoft, Tulsa, OK, USA, version Cz. 7). As most of the data did not meet standard criteria of normality, the difference between dog owners and non-dog owners in PA level, sleep, and perceived degree of health was evaluated using non-parametric tests (Mann–Whitney U test). To evaluate the relationship between selected variables, Spearman rank correlation was used. Results were considered statistically significant when *p* ≤ 0.05.

## 3. Results

### 3.1. General Results

Demographic data shows that from 44 total participants 26 older adults live in a household with another person, out of which 15 are dog owners; and 18 older adults live alone, out of which 11 are dog owners. The mean length of education for both groups together is 13 years. Table 1 shows basic characteristics of dog owners (DO) and non-dog owners (NDO).

The accelerometer measurement shows that on average all older adults included in the research took 8032 steps per day and 56,228 steps per week. The time spent on overall activity was 105 min per day on average, and 735 min per week. The distance they walked during the day was an average of 5.4 km per day (37.9 km per week). During this physical activity, they released 164 kcal a day. Weekly it was 1147 kcal. The total sleep duration of older adults was 418 min/night on average, weekly 2915 min/night. The mean deep sleep length of an older adult was 131 min/night and weekly 900 min/night. The mean light sleep length of both groups together was 289 min/night, weekly 2012 min/night. The time when older adults were awake at night was measured 16 min/night and weekly 112 min/night. Table 2 shows comparison of this data between dog owners and non-dog owners.

The IPAQ-SF questionnaire shows that older adults weekly (the whole group, DO + NDO) performed on average 32 min of vigorous physical activity (735 MET-mins/week), 65 min of moderate physical activity (596-MET mins/week), and they spent on average 116 min (2499 MET-mins/week) walking. Table 3 shows comparison of the above-mentioned data between DO and NDO.

The results of the SF-36 questionnaire showed that the self-reported health of older adults was on average as follows: general health 61%; health change 45%; physical functioning 81%; role of limitations due to physical health 79%; role of limitations due to emotional problems 85%; social functioning 84%; pain 73%; vitality 59%; and emotional well-being 75%. Table 4 shows again comparison of this data between DO and NDO.

### 3.2. Results of Dog Owners vs. Non-Dog Owners

#### 3.2.1. Accelerometer—Step Count, Activity Time, Distance, and Calorie Count

When comparing DO and NDO groups, a statistically significant difference was observed in favor of dog owners in all of the monitored parameters. All parameters showed a *p* < 0.01 (activity time—*p* = 0.0001; calorie count—*p* = 0.001; step count—*p* = 0.0003; distance—*p* = 0.0006) (Table 2).

#### 3.2.2. Accelerometer—Sleep Qualities

The sleep evaluation results indicate that there is a statistically significant difference in sleep for the light sleep length (*p* < 0.05) between DO and NDO. Dog owners sleep an average of 307 min of light sleep at night, non-dog owners 273 min per night. The total length of sleep is at the borderline of statistical significance *p* = 0.05 in favor of DO.

The length of sleep correlates with SF 36 items only in the deep sleep category with the pain parameter. This correlation is positive (r = 0.37; *p* < 0.05).

#### 3.2.3. IPAQ

When comparing the values of both groups reported in the IPAQ questionnaire (Table 3), a statistically significant difference was observed in favor of dog owners in MET/min that older adults spent walking (*p* = 0.007). This also affected the total MET/min, which was significantly higher in older adults owning a dog (*p* = 0.002). Furthermore, dog owners burned more calories (*p* = 0.002) and their time spent performing all activities (vigorous + moderate + walking) was higher (*p* = 0.017).

A significant result was also the statistical difference (*p* = 0.017) between the groups in the classification according to the performed PA (high, medium, low). Older adults with a dog were more often categorized as belonging to “high”.

Different results were also seen in BMI assessment, where older adults in DO group had significantly lower BMI than older adults in NDO group (*p* = 0.02). The mean BMI for both groups together is 27.2 points. Dog owners have a lower BMI (26.1) than non-dog owners (28.8).

#### 3.2.4. SF 36

There was also a difference between groups in favor of dog owners in the SF-36 rating (Table 4). In this questionnaire DO older adults showed significantly better results in the evaluation of general health (*p* = 0.0000), physical functioning (*p* = 0.0009), social functioning (*p* = 0.0103), pain (*p* = 0.0159), vitality (0.00000), and emotional well-being (*p* = 0.0086). No statistical difference was observed when looking at the other parameters. However, the NDO group did not perform better than DO group in any of the monitored parameters of the SF-36 questionnaire.

## 4. Discussion

The results show that the whole set of older adults that we followed averaged fewer steps a day than a similar set of older adults in the Czech Republic, as reported in the study by Gába et al. [26]. When comparing the average daily number of DO steps with this study, however, our group (DO) exceeds the average population in the Czech Republic, while NDO reach only half of the steps.

However, comparing vigorous PA of our study to the results of the above-mentioned study brought contrary results. The whole set of our subjects achieved significantly higher VPA, which is caused by high VPA in DO while NDO have a comparable number of minutes a week to older adults from the Gába et al. study. On the contrary, both groups have significantly lower MPA than the population in the study of Gába et al.

Dall et al. [27] report very similar daily step count of dog owners, however, daily step count of non-dog owners is about 2000 steps lower. Additionally, time spent walking by the DO group is similar as in this study, but walking time of the NDO group is higher than in this study. Another study [28] supports possible effect of dog ownership on physical activity. VPA and MPA of our DO group were higher than VPA and MPA of NDO but it did not reach required statistical significance. Walking time measured in MET-mins was significantly different when comparing DO vs. NDO groups. Similarly, MET-mins walking time of DO in the mentioned study was higher than the NDO walking time, but when comparing with our data, it was same as our NDO group while our DO group highly exceeds the Mein et al. [28] DO group.

The results of accelerometer data indicate that, as in a similar study by Toohey et al. [29], the dogs had a positive effect on the physical activity of their owners when measuring PA with an accelerometer. The living environment of the participants (i.e., presence of a yard, fence, sidewalks) can have impact on the walking behaviors of participants. It is suggested that the dog walking can have a positive effect on overcoming problems associated with the surroundings. One reason may be the regularity of the routes that the individual creates. It might be interesting for future studies to focus on this phenomenon. On the one hand, in the regularity of walks the dog owner knows the route well and may feel more comfortable, on the other hand, it may deprive them of other routes where there would be different length and terrain.

It is interesting to compare the activity time of the accelerometer with walking data in the IPAQ questionnaire. Although it is a measurement of comparable parameters, the results show a significant difference between accelerometer and questionnaire data. It points to a certain limit of the IPAQ-SF questionnaire as reported by other studies [30,31,32,33]. This age group may experience problems recalling total physical activity over the past seven days, although cognitive functions do not show signs of dementia. However, the use of an accelerometer also has its limits [34,35]. One of them is the need to recharge the wristband and synchronize it with the smartphone.

The advantages of the Xiaomi Mi Band 2 accelerometer are its high measurement accuracy (96.56%), relatively low variation coefficient (CV = 5.81), long battery life (3–4 weeks), and low purchase price [19]. Furthermore, the sleep activity measurement feature is very convenient, although the authors are aware of the limitations associated with this device, such as the fact that this type of accelerometer specifically does not record sleep during the day. Another disadvantage of the accelerometer used in our study is that it counts only activity exceeding 4 min as the active time. It is therefore possible that the duration of the activity is slightly higher.

Just as in a study by Wood et al. [36] older adults owning a dog showed significantly better values in parameters related to the social area of SF-36 questionnaire. These results may be related to regular walking of the dog. Knight and Edwards [37] state that older adults deliberately choose places for walking their pets where they can meet other dog owners. Ownership of the dog and a strong relationship with it is a motive for communicating and having conversations about their pets. Walking with the dog facilitates contact with the environment and provides opportunities for new social ties. This form of social support is manifested not only in public, but also at home in the presence of friends and family. If an older adult does not have enough contact with loved ones, the dog can replace a family member for him or her [37] and thus give him or her desired social contact. The second important parameter in favor of dog owners was, as reported by Feng et al. [10], physical functioning. This fact, together with a higher number of minutes spent vigorous PA, indicates a higher physical fitness of dog owners. The higher number of steps dog owners take is thus positively reflected in the physical functioning of older adults.

An interesting result from the IPAQ questionnaire is the sedentary time. Sedentary values were almost the same between the dog owners and non-dog owners, which is contrary to the available research [11,38]. The review by Harvey et al. [39] points out that older adults more frequently report sedentary time in questionnaires between 4 and 5 h, while objectively measured accelerometer data show sedentary time more than 8.5 h per day. This difference also indicates the limits of the questionnaire that need to be considered. However, our data are comparable to other studies that also use IPAQ as a tool. The average time spent sitting was 6 h for both groups together, which is relatively low compared to other studies conducted abroad [33,38,40].

In this study, achieving recommended levels of PA can be compared from two different angles. For example, the World Health Organization [1] recommends 150 min of moderate physical activity or 75 min of vigorous physical activity. The data recorded by the accelerometer shows only the total activity time, i.e., the time of both vigorous and moderate physical activity and the time spent walking. It can be concluded that 735 min achieved by our group of older adults far exceeded the recommended level. The second way to answer this question is to compare the number of steps. In the review of Tudor-Locke and Myers [41], authors reviewed over 15 studies of physical activities in older adults and stated that the average number most healthy older adults achieve is 6000–8500 steps/day. A range of steps that is suitable for older ages and has a positive effect on the health of older adults is determined by Rowe et al. [42] as 8000–10,000 steps per day. The total number of steps of older adults in our study was 8032 steps/day. The daily number of steps of our group of older adults is thus equal to the number of steps of older adults in other countries.

Available studies indicate that owning a dog and having to walk it has a significant effect on overweight and obesity. Coleman et al. [43] reported a significantly lower percentage of obese owners who walk regularly compared to those who do not walk or do not own a dog. Thorpe et al. [44] also talked about lower weight of older adults who walked their dog. However, authors also stated that the people who owned a dog but did not walk it were about 12.5% more obese. This suggests that walking is the cornerstone of how dog ownership influences weight reduction. The results of our study support this statement; however, it must be noted that walking time and dog walking time were not separate measures in this research. However, as we assume from the study of Richards et al. [45] lower weight of DO is a result of dog ownership, not dog walking. It is important to state this, because some older adults may choose to not walk their dog, or they do not walk it enough. There are many factors that influence dog walkers in this decision, mainly relationship between the dog and its owner, social and family support, and neighborhood environment, like presence of yards and trails [46].

All respondents fell into the category of grade 1 overweight, which at the level of normal health of this age category corresponds to the assessed rate of physical activity and sitting time. The observed BMI supports the average values of foreign studies, where this figure is also around 28 while considering all possible factors [47,48,49].

An important question still remains unanswered whether more active people own dogs of whether the dogs make people more active. This cannot be determined with this type of study design but for sure dog walking can bring many positive effects to people and older adults seem to be a suitable target group that can profit from these benefits.

### Limits

The cross-sectional study design can be viewed as major drawback of our study because we can confirm group difference only and cannot prove causality. Thus, the results should be viewed with a certain degree of caution.

Another limit of this study is that participants were not a representative sample and it was not possible to examine potential moderating factors. Older adults were also participating based on their willingness, which could influence the results. We are aware of the fact that substantial limitation of this study can be selecting only dog owners who walk their dog. For future stronger study design it would be necessary to have also a group of dog owners who do not walk their dogs.

Another limitation was a subjective evaluation of the IPAQ Short form questionnaire of questioned persons. Older adults may have a problem recalling physical activity performed in the past week and therefore it can bias the result to a certain extent. There is also a disadvantage of the accelerometer used in our study is that it counts only activity exceeding 4 min as the active time. It is therefore possible that the duration of the activity is slightly higher. Additionally, sleep is measured only in night time, so daytime naps were not considered in the sleep parameters. Another limitation is the need to recharge the wristband and synchronize it with smartphone. Future studies could aim to optimize the measurement and synchronize available methods.

## 5. Conclusions

Physical activity is crucial for maintaining good health, both physical and mental. Thus, it is very important for people to perform enough physical activity, especially in the older population. Older adults who own a dog show significantly higher PA in both IPAQ-Short form results and in the values measured by the accelerometer, including sleep. A positive effect on their overall health assessed by SF-36 was observed in most of the monitored parameters. The results suggest that the dog walking affects the overall PA of older adults and it brings positive effects to the length of sleep and quality of life.

## Figures and Tables

**Table 1:** Demographic data of dog owners (DO) and non-dog owners (NDO).

	DO (*n* = 26)	NDO (*n* = 18)	*p*-Value
Sex (% of males)	0.54	0.22	0.0773
Living alone (%)	0.42	0.39	0.8486
Employed (%)	0.27	0.28	0.9619
Mean age ± SD (years)	68 ± 5.4	71 ± 5.5	0.0734
Mean length of education ± SD (years)	14 ± 3.3	12 ± 3.1	0.1124
Mean height ± SD (cm)	170.2 ± 6.7	166.3 ± 6.5	0.1662
Mean weight ± SD (kg)	75.8 ± 15.5	80.4 ± 21.5	0.3901
Mean BMI ± SD (kg.m^–1^)	26.1 ± 4.1	28.8 ± 5.4	0.0213

**Table 2:** Comparing the DO and the NDO using data from accelerometer, mean ± SD.

	DO	NDO	*p*-Value	Effect Size
Step count/day	9961 ± 5213	5247 ± 2644	0.0003	0.50
Activity time (min)/day	127 ± 62	73 ± 28	0.0001	0.50
Distance (km)/day	6.7 ± 3.8	3.5 ± 1.8	0.0006	0.47
Calories/day	200 ± 97	112 ± 60	0.0014	0.48
Sleep length (min)/day	447 ± 87	394 ± 86	0.0593	0.29
Light sleep length (min)/day	309 ± 50	273 ± 55	0.0401	0.32
Deep sleep length (min)/day	136 ± 50	124 ± 56	0.5192	0.11
Awake time at night (min)/day	13 ± 15	20 ± 21	0.2058	−0.18

**Table 3:** Comparing the DO and the NDO using data from IPAQ (International Physical Activity Questionnaire), mean ± SD.

	DO	NDO	*p*-Value	Effect Size
VPA (min)/week	50 ± 70	8 ± 29	0.0610	0.36
MPA (min)/week	73 ± 52	52 ± 49	0.1774	0.20
Walking (min)/week	128 ± 48	99 ± 58	0.0661	0.26
Total PA (min)/week	252 ± 126	158 ± 80	0.0170	0.41
Sitting (min)/week	353 ± 125	363 ± 142	0.8113	−0.04
VPA (MET-min)/week	1123 ± 1847	173 ± 678	0.0643	0.32
MPA (MET-min)/week	700 ± 589	447 ± 619	0.0879	0.20
Walking (MET-min)/week	2910 ± 1114	1904 ± 1143	0.0075	0.41
Total PA (MET-min)/week	4733 ± 2671	2524 ± 1557	0.0021	0.45
Calories/week	6032 ± 3759	3186 ± 1876	0.0020	0.43
PA level (n)				
High	16	5		
Moderate	10	9		
Low	0	4		

**Table 4:** Comparing the DO and the NDO taken from SF-36. Data are presented in percentage (mean ± SD).

	DO	NDO	*p*-Value	Effect Size
General health	72 ± 15	46 ± 14	0.0000	0.67
Health change	47 ± 11	43 ± 14	0.3771	0.16
Physical functioning	88 ± 12	72 ± 22	0.0009	0.41
Role of limitations due to physical health	85 ± 27	71 ± 33	0.1555	0.23
Role of limitations due to emotional problems	86 ± 29	83 ± 26	0.6416	0.05
Social functioning	90 ± 18	76 ± 18	0.0103	0.36
Pain	78 ± 19	62 ± 22	0.0159	0.36
Vitality	67 ± 15	47 ± 6	0.0000	0.66
Emotional well-being	80 ± 12	69 ± 13	0.0086	0.40
